# Enriching a primary health care version of ICD-10 using SNOMED CT mapping

**DOI:** 10.1186/2041-1480-1-7

**Published:** 2010-06-17

**Authors:** Mikael Nyström, Anna Vikström, Gunnar H Nilsson, Hans Åhlfeldt, Håkan Örman

**Affiliations:** 1 Department of Biomedical Engineering, Linköpings universitet, SE-581 85 Linköping, Sweden; 2 Department of Neurobiology, Care Sciences and Society, Center for Family and Community Medicine, Karolinska Institutet, SE-141 83 Huddinge, Sweden

## Abstract

**Background:**

In order to satisfy different needs, medical terminology systems must have richer structures. This study examines whether a Swedish primary health care version of the mono-hierarchical ICD-10 (KSH97-P) may obtain a richer structure using category and chapter mappings from KSH97-P to SNOMED CT and SNOMED CT's structure. Manually-built mappings from KSH97-P's categories and chapters to SNOMED CT's concepts are used as a starting point.

**Results:**

The mappings are manually evaluated using computer-produced information and a small number of mappings are updated. A new and poly-hierarchical chapter division of KSH97-P's categories has been created using the category and chapter mappings and SNOMED CT's generic structure. In the new chapter division, most categories are included in their original chapters. A considerable number of concepts are included in other chapters than their original chapters. Most of these inclusions can be explained by ICD-10's design. KSH97-P's categories are also extended with attributes using the category mappings and SNOMED CT's defining attribute relationships. About three-fourths of all concepts receive an attribute of type *Finding site *and about half of all concepts receive an attribute of type *Associated morphology*. Other types of attributes are less common.

**Conclusions:**

It is possible to use mappings from KSH97-P to SNOMED CT and SNOMED CT's structure to enrich KSH97-P's mono-hierarchical structure with a poly-hierarchical chapter division and attributes of type *Finding site *and *Associated morphology*. The final mappings are available as additional files for this paper.

## Background

### Medical terminology systems evolution

There are various types of medical terminology systems to satisfy different needs. To satisfy more needs than what exist today, both Rossi Mori et al. [1] and Cimino [2] ask for an evolution of the medical terminology systems for more flexiblity.

Rossi Mori et al. describe three generations of medical terminology systems [1]. The *first generation *comprises traditional terminology systems [1]. This generation includes controlled vocabularies, nomenclatures, taxonomies and coding systems which satisfy most needs in paper-based information systems. In this generation, systems typically consist of a *list of phrases*, a *list of codes*, a *coding scheme *and a *hierarchy*. The role of the coding scheme is to map between phrases and codes [1]. Examples of systems in the first generation are ICD-10, KSH97-P and International Classification of Functioning, Disability and Health (ICF).

The *second generation *are compositional systems. These systems have a *categorical structure*, *a cross-thesaurus*, a *structured list of phrases *and a *knowledge base of dissections *[1]. The categorical structure gives a high-level description of the content, i.e. what kinds of concepts are included and how they relate to each other. This can be seen as a framework of slots for which the cross-thesaurus provides a set of labels to be inserted when the content is modelled. By means of the cross-thesaurus, each element in the structured list of phrases is represented according to the categorical structure; these descriptions constitute the knowledge base of dissections. Examples of systems in the second generation are Nomenclature, Properties and Units (NPU), Logical Observation Identifiers, Names and Codes (LOINC), and SNOMED International [1].

The *third generation *consist of formal systems. In this generation, the systems have a *set of symbols *and a *set of formal rules *to manipulate the symbols and these sets can be seen as a set of concepts and a set of relations between the concepts [1]. It is possible to represent each concept in a unique canonical form and a non-canonical expression may be automatically converted to a unique canonical form using an engine. An example of a third generation system is GALEN-IN-USE's surgical procedures [1]. SNOMED CT is evolving towards a third generation system.

One problem in the first generation is that reorganisation of categories in the systems to satisfy different purposes is not supported [1]. Reuse of data organised with first generation systems therefore needs human interpretation of the categories and the environment where the data was originally collected. This problem is smaller in the second generation and even smaller in the third generation. In the second generation, categories can be reorganised according to the information in the knowledge base of dissection. In the third generation, formal rules can be used for reorganisation [1].

Cimino enumerates twelve characteristics of the structure and content in medical terminology systems in "Desiderata for controlled medical vocabularies in the twenty-first century" and these characteristics emerge from earlier vocabulary research [2]. Four of these characteristics are relevant in this study

• *Poly-hierarchy*. Systems need to shift from a strict mono-hierarchy (taxonomy) to a poly-hierarchy [2]. It is impossible to properly represent the real world in a strict mono-hierarchy where each category has only one parent. The categories in the real world can belong to more than one parents [2-5].

• *Formal definitions*. Systems need formal definitions expressed as collections of different kinds of relationships between the concepts [2]. Formal definitions can be used by computers for formal manipulations of the categories which is impossible with unstructured text definitions [2]. One manipulation is to help a user locate a specific category in a terminology system [3]. A similar manipulation is locating where to include new categories in the system's structure [3-5]. Another manipulation is to test whether a pre-coordinated category is equivalent to a set of post-coordinated categories or whether different sets of post-coordinated categories are equivalent [5].

• *Multiple granularity*. Systems need to have concepts of different granularity covering the same area [2]. Different use cases need systems with different granularities depending on the required level of detail of the categories. A multipurpose system therefore needs multiple granularities [2]. One use case is abstracting information in health records to allow compilations of health records' contents. Another use case represents sufficient detail of the information in health records in order to use the information in for example direct patient care, decision support and quality assurance [5].

• *Multiple consistent views*. Systems need to be able to consistently present their content in different views [2]. Some use cases require a simple structure of the system's categories and others need a richer structure. The kind of structure depends on the required level of detail and required type of information of the categories [2,3]. To present equivalent information independent of the view used, the views need to be consistent [2,3].

### Information reduction using medical terminology systems

Straub et al. have a different opinion than Rossi Mori et al. and Cimino as presented above [6]. They argue that the different kinds of medical terminology systems have different purposes and need to co-exist. Medical terminology systems with fewer categories and a semantic model with more restrictions, such as a hierarchical tree, provide useful information reduction or simplification for cases where a richer medical terminology system provides too much information [6].

Hierarchical trees are disjunctive and unidirectional [6]. Disjunctive means that all categories on one level are mutually exclusive and unidirectional signifies all hierarchical relations only go in one direction. Unfortunately, diseases are not disjunctive and unidirectionally related to each other and therefore it is not possible to construct a hierarchical tree of diseases based on the diseases' characteristics [6]. If a hierarchical tree is still constructed from disease categories, for which Straub et al. think there are good reasons, the hierarchical tree is artificial. The drawback of artificial hierarchical trees is the structure is arbitrary. This means that in the construction of the tree, some information is hidden -- the information on which the hierarchy is not based [6].

### Modelling of health problems

ICD-10 [7] is primarily intended for statistical reporting and administrative tasks such as disease monitoring and quality assurance [8]. Although neither based on nor intended as a model of health problems, but pragmatically developed from the admittedly arbitrary structure proposed by William Farr in 1855 [9,10], the ICD classifications are by far the most used terminology systems in electronic health records [11].

Farr's structure, which is reflected in how diseases are divided into chapters in the ICD-10, groups diseases into five sets [9,10]:

• epidemic diseases

• constitutional or general diseases

• local diseases arranged by site

• developmental diseases

• injuries

The presentation of ICD-10 [7,9] focuses on the role as a member of a family of classifications rather than the internal structure. In ICF, one part of the introduction describes a conceptual framework for the classification [12]; that kind of model does not exist for ICD-10.

While ICD classifications are mono-hierarchical, the International Classification of Primary Care (ICPC) [13], originally published in 1987 and later in a second [14] and a revised second edition [15], is bi-axial, consisting of chapters and components. Here, a patient's reason for encounter, health problems to be taken care of and interventions are classified and coded according to a chapter structure. The chapter structure is based on body systems and problem areas and a set of components specifying the nature of the phenomenon coded such as a complaint, procedure or disease.

The move towards the third generation of terminology systems with formal definitions of disorders has proven to be a challenging task [16]. This is especially valid if diagnostic criteria are to be taken into account as is the case for psychiatric diagnoses in the Diagnostic and Statistical Manual for Mental Disorders (DSM) as well as ICD [17-20]. Version 3 of the Read Codes, a constituent of SNOMED CT, presented a template-based mechanism with attributes and values for basic semantic operations on items [21,22]. A set of categories describing completeness of definitions was developed as a by-product in the process of disorder definition [21]. However, Version 3 of the Read Codes is still a second generation terminology system [1].

Héja et al. have presented work on formal definitions of the ICD-10 based on the GALEN [23] and DOLCE [24] formalisms with the main objective of providing a knowledge-based coding support tool. They found that although lexical processing [23] as well as existing terminology resources [24] may assist formal representation, ICD categories themselves--owing to the historical development rooted in epidemiological considerations--deviate from what is expected in contemporary ontology engineering [23]. The result is a need to distinguish the meaning of categories from the structure of the classification, which essentially was the underlying rationale in the early modelling work reported by Petersson et al. [25]. Such pitfalls of pragmatic classifications have also been reported in surgery, an area that modelling-wise is usually considered more straightforward than the domain of diseases [26].

Alecu et al. created a grouping of the categories in the World Health Organisation - Adverse Reaction Terminology (WHO-ART) based on mappings between WHO-ART and SNOMED CT in the Unified Medical Language System (UMLS) Metathesaurus [27]. More specifically, they used synonym relations between WHO-ART categories and SNOMED CT concepts, creating synonym relations for 85.9% of all categories.

As pointed out by Rossi Mori et al. [1], and demonstrated by Alecu et al. [27], second and third generation systems can augment first generation systems with easier re-organisation and maintenance and with harmonisation and cross-referencing of different first generation systems. The description of the categorical structure could also be used for systematic comparison of terminology systems such as ICD, ICPC and SNOMED CT. Ingenerf and Giere argue along the same line when they explore the different roles of statistical classifications and formal concept representation systems, deducing the need for co-existence and the former being linked to the latter [28]. The empirical results described above indicate these merely theoretical assertions require considerable thought before they are realised, which is consistent with the finding that little evidence, other than theoretical, exists on the usefulness of SNOMED in clinical practice [29].

### Objective

The primary health care terminology system "Klassifikation av sjukdomar och hälsoproblem 1997 Primärvård" (KSH97-P) is based on the International Statistical Classification of Diseases and Related Health Problems, Tenth Revision (ICD-10). The general objective is to explore whether mappings from KSH97-P to SNOMED CT and SNOMED CT's structure can be used to enrich KSH97-P's mono-hierarchical structure. The enrichment thereby hypothetically provides useful multiple views of a disease panorama as coded with a traditional disease classification. The objective contrasts with the related work by Héja et al. [23,24] presented above, where the objectives were to develop a new formal concept representation system of ICD categories, but is in line with the intentions of Alecu et al. [27]. The results are discussed in relation to improvements of medical terminology systems as presented in the background.

The first specific question is whether SNOMED CT's poly-hierarchical generic structure can be used to add a multiple chapter division to KSH97-P's categories where each category may belong to more than one chapter. The second specific question is whether SNOMED CT's defining attribute relationships can be used to add attributes to KSH97-P's categories.

## Methods

A glossary with explanations of the used terms is included at the end of this paper.

### SNOMED CT

SNOMED CT is a clinical terminology intended for clinical documentation and reporting [30]. In other words, SNOMED CT covers both abstraction and representation [5]. It consists of *concepts*, *descriptions *and *relationships *[30].

Here, a concept is a clinical meaning and is identified by a unique number. Associated with each concept are two or more descriptions, which are human readable terms, and information about the terms [30].

Relationships link concepts to each other and are of different *relationship types *[30]. The generic relationship type *Is a *relates *subtypes *to *supertypes *and is always a *defining relationship*. All concepts, except for the root concept, have at least one *Is a *relation to a supertype concept [30]. The other relationship types that are defining relationships are the *defining attribute relationships*. The defining relationships logically represent a concept by establishing relationships between the concepts [30].

A concept in SNOMED CT can either be *fully defined *or *primitive *[30]. A fully defined concept is modelled as described above so it is possible to distinguish the concept from the other concepts through its relationships with other concepts. Primitive concepts lack one or more relationship(s) to be able to fully distinguish from other concepts using the concept's relationships [30]. There is also a concept model that controls which types of concepts can be related to which types of relations [30].

Concepts in SNOMED CT can be retired from active to inactive concepts [30]. Inactive concepts have historical relationships that relate the inactive concepts to active concepts. The historical relationships can be used to point out active concepts that replace inactive concepts [30].

### KSH97-P

The Swedish National Board of Health and Welfare has worked out a primary health care version of the International Statistical Classification of Diseases and Related Health Problems (ICD-10) [31] (in Swedish, *Klassifikation av sjukdomar och hälsoproblem 1997 Primärvård (KSH97-P)*). Codes and rubrics (in both Swedish and English) of KSH97-P together with mappings to ICD-10 can be downloaded from their Web site [32].

KSH97-P contains 972 categories which concern diseases and health-related problems common in primary health care [31]. Most categories in KSH97-P correspond to categories in the three or four-character levels in ICD-10. Some categories in KSH97-P correspond to two or more similar categories in ICD-10. Some categories in ICD-10 which are less frequently used in primary health care have been merged with related unspecified categories in ICD-10 to corresponding categories with broader coverage in KSH97-P [31]. Rubrics in KSH97-P are as close to the Swedish translation of ICD-10 as possible [31].

Examples of KSH97-P categories and corresponding ICD-10 categories are [31]

• KSH97-P category A00- *Cholera *corresponds to the ICD-10 category A00 *Cholera*.

• KSH97-P category J45-P *Asthma *corresponds to the two ICD-10 categories J45 *Asthma *and J46 *Status asthmaticus*.

• KSH97-P category H669P *Otitis media, unspecified *corresponds to the ICD-10 category H66.4 *Suppurative otitis media, unspecified*, which is more specific than the category in KSH97-P. H669P also corresponds to the ICD-10 category H66.9 *Otitis media, unspecified*, which is equally specific as the category in KSH97-P.

KSH97-P has the same chapter division as ICD-10. The exceptions are that ICD-10 chapter XX *External causes of morbidity and mortality *is left out from KSH97-P [31] and chapter XXII *Codes for special purposes *is left out in both the Swedish version of ICD-10 [33] and KSH97-P [31]. The rubric and number of categories in each chapter are included in Table 1.

**Table 1:** KSH97-P and KSH97-P mappings

Chapter	Name	Number of categories	Number of mapped categories	Mapped chapter	Number of categories excluded in multiple chapter division
I	Certain infectious and parasitic diseases	83	83	Yes	1

II	Neoplasms	79	79	Yes	0

III	Diseases of the blood and blood-forming organs and certain disorders involving the immune mechanism	13	13	Yes	1

IV	Endocrine, nutritional and metabolic diseases	30	30	Yes	4

V	Mental and behavioural disorders	47	47	Yes	2

VI	Diseases of the nervous system	35	35	Yes	3

VII	Diseases of the eye and adnexa	36	36	Yes	1

VIII	Diseases of the ear and mastoid process	21	21	Yes	2

IX	Diseases of the circulatory system	50	49	Yes	4

X	Diseases of the respiratory system	38	38	Yes	0

XI	Diseases of the digestive system	49	48	Yes	5

XII	Diseases of the skin and subcutaneous tissue	64	63	Yes	5

XIII	Diseases of the musculoskeletal system and connective tissue	83	81	Yes	17

XIV	Diseases of the genitourinary system	66	65	Yes	11

XV	Pregnancy, childbirth and the puerperium	30	29	Yes	1

XVI	Certain conditions originating in the perinatal period	16	16	Yes	0

XVII	Congenital malformations, deformations and chromosomal abnormalities	40	40	Yes	2

XVIII	Symptoms, signs and abnormal clinical and laboratory findings not elsewhere classified	93	91	No	70

XIX	Injury, poisoning and certain other consequences of external causes	64	64	Yes	11

XXI	Factors influencing health status and contact with health services	35	30	No	34

Σ		972	958	18	174

The KSH97-P chapters' names, the number of categories in each chapter, the number of categories in each chapter that are mapped to SNOMED CT, whether a chapter is mapped to SNOMED CT or not and the number of categories in each chapter that are not included in the multiple chapter division.

KSH97-P mixes categories related to ICD-10 categories in both three and four-character levels. Therefore, the National Board of Health and Welfare recommends to only compile statistics on the chapter level or to use customised groups of categories [31].

As described above, ICD-10, and thus KSH97-P [31], uses multiple principles for chapter division [7]. Some chapters contain categories related to a specific organ system and other chapters contain diseases with some specific aetiology. There are also chapters containing categories related to pregnancy, childbirth and the puerperium; the perinatal period; symptoms and partially specified cases; and important factors for contact with the health care system [7]. The preface to KSH97-P states these different kinds of chapter divisions may imply practical problems because it is not evident to which chapter a specific disease or condition belongs [31].

In ICD-10, and thus KSH97-P [31], a category can be only included in one chapter [7]. For those categories in which it would be possible to include more than one chapter, a decision has been made about into which chapter to include the category. This is demonstrated in ICD-10 by the excludes remarks on the chapter level. An excludes remark means that the categories in the remark could have been included in the chapter, but are instead included in other specified chapters [7]. Table 2 summarises the excludes remarks on the chapter level for three-character level exclusions [7]. The excludes remarks for four-character level exclusions on the chapter level are omitted because they only contain six categories [7].

**Table 2:** Exclusions of three-character categories in ICD-10

Chapter	Priority																					
	
	I	II	III	IV	V	VI	VII	VIII	IX	X	XI	XII	XIII	XIV	XV	XVI	XVII	XVIII	XIX	XX	XXI	XXII
I										.30					.01	.08					.01	

II																						

III	.03	1.00		1.00											1.00	1.00	1.00	1.00	1.00			

IV															1.00	.07		1.00				

V																		1.00				

VI	1.00	1.00		1.00											1.00	1.00	1.00	1.00	1.00			

VII	1.00	1.00		1.00											1.00	1.00	1.00	1.00	1.00			

VIII	1.00	1.00		1.00											1.00	1.00	1.00	1.00	1.00			

IX	1.00	1.00		1.00		.01							.09		1.00	1.00	1.00	1.00	1.00			

X	1.00	1.00		1.00											1.00	1.00	1.00	1.00	1.00			

XI	1.00	1.00		1.00											1.00	1.00	1.00	1.00	1.00			

XII	1.00	1.00		1.00									.09		1.00	1.00	1.00	1.00	1.00			

XIII	1.00	1.00		1.00											1.00	1.00	1.00	1.00	1.00			

XIV	1.00	1.00		1.00											1.00	1.00	1.00	1.00	1.00			

XV	.04				.01														1.00		.02	

XVI	.01	1.00		1.00													1.00		1.00			

XVII				.26																		

XVIII																						

XIX															.03	.10						

XX																						

XXI																						

XXII																						

The exclusions of three-character categories on the chapter level according to the excludes remarks at the beginning of each chapter in ICD-10 [7] are summarised in this table. The table shows the proportion of categories in the ICD-10 chapters in the columns that have priority over the ICD-10 chapters in the rows according to the excludes remarks.

An example is that 26% of the three-character categories in chapter IV have priority over chapter XVII.

### Three-dimensional structure of KSH97-P

To transform KSH97-P from a first generation system to a second generation system, a three-dimensional additional structure was added to KSH97-P in a previous research project [34]. In the three-dimensional structure, each category was categorised according to location, origin and type [25].

### Baseline category mapping

A baseline category mapping from KSH97-P's categories to SNOMED CT's concepts is used. The first phase of the mapping process is described in a reliability study where mapping was done by two coders [35]. KSH97-P was randomly divided into three sets of categories, used in three mapping sequences. Mapping was done independently by the coders and mapping rules were developed and agreed upon between the sequences. In the last round, mapping was completed through consensus decisions, following the mapping rules and striving to achieve a result with "completely concordant" mappings for each category. In the mapping, disorder and finding concepts were given priority and there was no use of navigational concepts [35]. The version used was the releases of SNOMED CT from January and July 2006. In summary, 14 (1%) of the 972 categories in KSH97-P did not have a matched concept in SNOMED CT, 888 (91%) were mapped to one concept, 64 (7%) were mapped to two concepts, and 6 (1%) were mapped to three concepts. Of the 958 mapped categories, 938 (98%) categories were mapped to clinical finding concepts and 20 (2%) categories were mapped to procedural concepts.

Examples of baseline category mappings are

• KSH97-P category A00- *Cholera *is mapped to the SNOMED CT clinical finding concept *Cholera*.

• KSH97-P category R252 *Cramp and spasm *is mapped to the SNOMED CT clinical finding concept *Cramp *and the clinical finding concept *Spasm*.

• KSH97-P category D38- *Neoplasm of uncertain or unknown behaviour of middle ear and respiratory and intrathoracic organs *is mapped to the SNOMED CT clinical finding concept *Neoplasm of intrathoracic organs *and the clinical finding concept *Neoplasm of middle ear *and the clinical finding concept *Neoplasm of respiratory tract*.

• KSH97-P category Z000 *General medical examination *is mapped to the SNOMED CT procedure concept *General examination of patient*.

### Methods used

**Figure 1:**
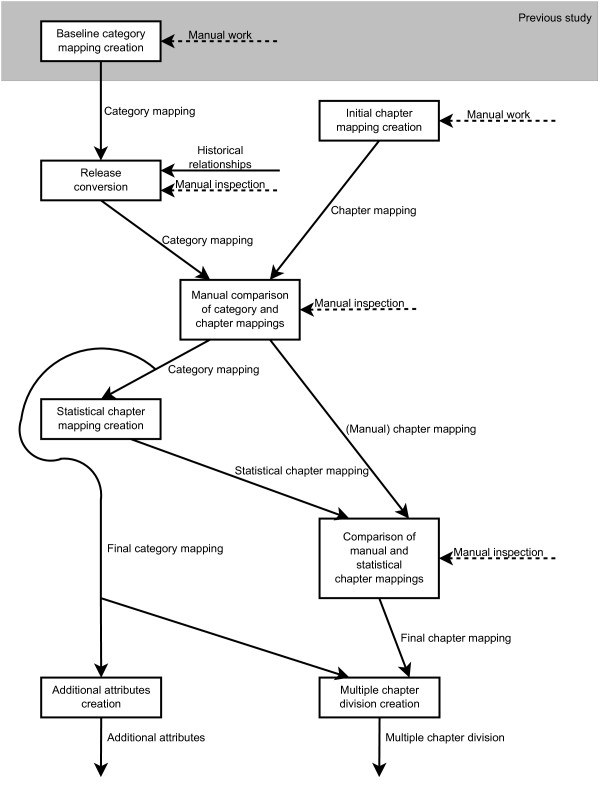
**Methods flowchart**. This figure illustrates how the methods used in this study are linked to each other. Except for the inputs shown in the figure KSH97-P and SNOMED CT's concepts, relationships and descriptions are used in all methods. The methods build upon the baseline category mapping that was manually built in the previous study [35]. The initial chapter mapping is manually built in this study and the baseline category mapping is converted to the same SNOMED CT version as the initial chapter mapping. The category mapping and chapter mapping are put together and are manually compared to each other and the mappings are updated. The category mapping is used to create the statistical chapter mapping. The statistical chapter mapping and the manually created chapter mapping are manually compared to each other and the manual chapter mapping is updated. The chapter mapping and category mapping are used for creating the multiple chapter division and the category mapping are used for creating the additional attributes.

A flow chart of the used methods is presented in Figure 1.

### Initial chapter mapping

Our study also needs a mapping from KSH97-P's chapters to SNOMED CT's concepts. The initial chapter mapping is therefore constructed during this study by the same persons mentioned above (Vikström et al. [35]).

The chapters are mapped to SNOMED CT's concepts based on the meaning of the chapter's rubric and a general assessment of both the chapter's content in ICD-10, using the international WHO-version of ICD-10 [7], and the subset of categories present in each chapter in KSH97-P. The same rules used for the category mapping and the excludes remarks in ICD-10 are considered as rules that do not exist in SNOMED CT. An example of an excludes remark is *certain localized infections *that should not be included in chapter I *Certain infectious and parasitic diseases *in ICD-10. Adequate mapping demanded good concordance between the rubric's meaning and the concept's meaning in SNOMED CT. For example, *Neoplastic disease *is considered a good match for chapter II *Neoplasms*. A concept could be considered as a reasonable match although it does not have relations to all categories from a certain chapter or it has relations to some categories from another chapter. An example is *Obesity *that is in chapter IV of ICD-10 but is not related to any of the mapped concepts in SNOMED CT as it is located directly under the *Disease *concept. The mapping is made to the SNOMED CT release January 2007.

Chapters XVIII *Symptoms, signs and abnormal clinical and laboratory findings, not elsewhere classified *and XXI *Factors influencing health status and contact with health services *are assessed as unable to map to SNOMED CT's concepts. The combination of different symptoms and abnormal clinical and laboratory findings in chapter XVIII's rubric are not considered to be a clinical concept, but a collection of different phenomena in a rubric that do not map to a concept or post-coordinated expression of manageable size in SNOMED CT. *Not elsewhere classified *is generally difficult to map to SNOMED CT, because the negation entails that the meaning of all other KSH97-P chapters has to be excluded in the mapped SNOMED CT concepts. Chapter XXI's rubric is likewise considered difficult to interpret as a clinical concept that could be mapped to a concept or post-coordinated expression of manageable size in SNOMED CT. This is especially due to the combination with the many categories in the chapter that describe procedures, not conditions and factors.

In summary, 2 (10%) of the 20 chapters in KSH97-P did not have a matched concept in SNOMED CT, 14 (70%) were mapped to one concept, 1 (5%) was mapped to two concepts, 2 (10%) were mapped to three concepts, and 1 (5%) was mapped to four concepts.

Examples of initial chapter mappings are

• KSH97-P chapter I *Certain infectious and parasitic diseases *is mapped to the SNOMED CT concept *Infectious disease*.

• KSH97-P chapter XIII *Diseases of the musculoskeletal system and connective tissue *is mapped to the SNOMED CT concept *Disorder of musculoskeletal system *and the concept *Disorder of connective tissue*.

### Mappings update

To improve the baseline category mapping and initial chapter mapping, the mappings are converted to the same SNOMED CT release, the category mappings and the chapter mappings are compared manually and statistical chapter mappings are calculated and compared with the manual mappings. All these steps are described below.

#### Release conversion

To be able to use only one release of SNOMED CT in this study, the baseline category mapping is transformed to SNOMED CT release January 2007 UK Edition. The transformation is done by keeping mappings to active concepts. For mappings to inactive concepts, a manual inspection of SNOMED CT's historical relationships from inactive concepts to active concepts is performed. When the manual inspection shows that the historical relationships replace the inactive concepts with suitable active concepts, then the active concepts are used for the new mapping. When the active concepts are not suitable for the mappings, new mappings are constructed manually.

Examples of updates during the release conversions are

• KSH97-P category E108P *Insulin-dependent diabetes mellitus with complications *has its map updated from the inactive SNOMED CT concept *Type I diabetes mellitus with complication *to the active concept *Disorder associated with type I diabetes mellitus *using the historical relationships.

• KSH97-P category M549P *Dorsalgia NOS *has its map updated from the inactive SNOMED CT concept *Back pain *to the active concept *Backache *using the historical relationships.

#### Manual comparison of category and chapter mappings

To check that the category and chapter mappings are not unintentionally mapped to different hierarchies in SNOMED CT, the category and chapter mappings are compared as described below.

SNOMED CT concepts to which any of the chapter mappings maps are collected in a set. For each concept in the set, the concepts' descendants are recognised and added to the set. The categories which do not map to any of the concepts in the set are then manually inspected. During the manual inspection, the categories' mappings are inspected together with relevant chapter mappings and the categories' and chapters' mappings are updated if suitable.

An example of an update during the manual comparison of category and chapter mappings is

• KSH97-P chapter II *Neoplasms *has its map updated from the SNOMED CT concept *Neoplastic disease *to the concept *Neoplasm and/or hamartoma *to better cover the categories in the chapter.

#### Statistical chapter mapping

A statistical chapter mapping is created for comparison with the manual chapter mapping. The statistical chapter mapping prefers concepts where the descendants are targets of many categories in the same chapter but few categories from other chapters. The creation of the mapping is described below.

The statistical chapter mapping is based on two quantities calculated for each combination of a chapter in KSH97-P and a concept in SNOMED CT (*n *times *m *possible instances, where *n *is the number of chapters and *m *is the number of concepts):

• *categories current chapter *(*c*): the number of categories in the current KSH97-P chapter that are mapped to the current SNOMED CT concept or any of its descendants.

• *categories other chapters *(*o*): the number of categories in other chapters than the current KSH97-P chapter that are mapped to the current SNOMED CT concept or any of its descendants.

For each combination of a chapter in KSH97-P and a concept in SNOMED CT where *c *> 0, the following score is calculated:

In other words, the calculations above determine the number of "correct" categories weighted with the compactness of "correct" categories in proportion to all categories.

For each chapter in KSH97-P, all SNOMED CT concepts are then ranked. The concept with the highest score is ranked as the best statistical chapter mapping and the concept with the second highest score is ranked as the second best statistical chapter mapping et cetera.

Examples of statistical chapter mappings are

• For KSH97-P chapter I *Certain infectious and parasitic diseases*, the best statistical chapter mapping is mapped to the SNOMED CT concept *Infectious disease*, the second best to *Bacterial infectious disease*, the third best to *Infection by site*, the fourth best to *Viral disease *and the fifth best to *Disease due to Gram-negative bacteria*.

• For KSH97-P chapter XVIII *Symptoms, signs and abnormal clinical and laboratory findings, not elsewhere classified*, the best statistical chapter mapping is mapped to the SNOMED CT concept *Clinical history and observation findings*, the second best to *General finding of observation of patient*, the third best to *Clinical finding*, the fourth best to *Neurological finding *and the fifth best to *Finding by method*.

#### Comparison of manual and statistical chapter mappings

A further check that the category and chapter mappings are not unintentionally mapped to multiple hierarchies in SNOMED CT is performed by comparison of the manual chapter mapping and the statistical chapter mapping as described below.

For each chapter, the manual chapter mapping is compared with the statistical chapter mappings. If a highly ranked statistical chapter mapping subsumes more of the concepts mapped from categories in the chapter than the manual mapping and the statistical chapter mapping is in line with the mapping rules, then the manual mapping is updated.

### Final mappings

The final mappings, which are the results of the mapping updates described above, are used in the rest of this study. The final category mapping is included as Additional file 1 and the final chapter mapping is included as Additional file 2. A summary of KSH97-P and the final mappings is included in Table 1.

### Multiple chapter division

To examine whether the poly-hierarchical *Is a *relationships of SNOMED CT can be used to replace KSH97-P's mono-hierarchical chapter division with a poly-hierarchical chapter division, KSH97-P's categories are divided into a multiple chapter division using SNOMED CT's *Is a *relationships. The multiple chapter division is generated using the algorithm described and exemplified below.

For each category, the mapped concepts are extracted together with their ancestors to a *mapped set*. This creates one mapped set for each mapped KSH97-P concept. If one or more chapters are mapped to any of the concepts in the mapped set, the category related to the mapped set is assumed to belong to these chapter(s)--regardless of what chapter they originally belong to. This means that each category may belong to zero, one or more new chapter(s).

**Figure 2:**
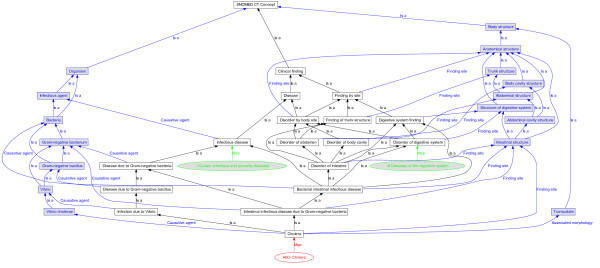
**Multiple chapter division algorithm and additional attributes algorithm example**. This figure illustrates how the multiple chapter division algorithm and the additional attributes algorithm are applied for the KSH97-P category A00- *Cholera*. The descriptions of the algorithms are included in the sections Multiple chapter division and Additional attributes. Several concepts, unimportant for the examples, are left out from the figure to decrease its size.

In the example below, the multiple chapter division algorithm is applied to the category A00- *Cholera*. The algorithm is illustrated in Figure 2.

The algorithm begins by creating a mapped set from an empty set. First, the category A00- *Cholera*, which is shown as a red ellipse in Figure 2, and its mapping are used to locate the concept(s) the category is mapped to. The algorithm finds that the category A00- *Cholera *is mapped to the concept *Cholera *and the concept *Cholera *is therefore added to the mapped set. All ancestors to the concept *Cholera *are then also added into the mapped set. The resulting mapped set consists of the concepts shown in black rectangles in Figure 2.

The algorithm then uses the mapped set to evaluate if any chapter(s) maps to any concept(s) in the mapped set. The algorithm finds that chapter I *Certain infectious and parasitic diseases *maps to the concept *Infectious disease *in the mapped set, and chapter XI *Diseases of the digestive system *maps to the concept *Disorder of digestive system *in the mapped set. The category A00- *Cholera *is therefore assumed to belong to the chapters I *Certain infectious and parasitic diseases *and XI *Diseases of the digestive system *according to the multiple chapter division. These chapters are shown as green shaded ellipses in Figure 2.

### Additional attributes

To examine whether the defining attribute relationships of SNOMED CT can extend KSH97-P categories with attributes, a list of additional attributes is created. The additional attributes are generated using the algorithm described and exemplified below.

For each category the mapped concepts are extracted together with their ancestors to a *mapped set*. This creates one mapped set for each mapped KSH97-P concept. (The mapped sets are created in the same way as for the multiple chapter division.) Then all defining attribute relationships from concepts in the mapped set are followed and the target concepts are included in a specific *attribute value set *for each relationship type. In each attribute value set, the concepts that are ancestors of another concept in the same attribute value set are removed. The remaining concepts in each attribute value set constitute attribute values of the respective attribute types for that category.

In the example below, the additional attributes algorithm is applied to the category A00- *Cholera*. The algorithm is illustrated in Figure 2.

The algorithm begins by creating a mapped set from an empty set. First, the category A00- *Cholera*, which is shown as a red ellipse in Figure 2, and its mapping are used to locate the concept(s) the category is mapped to. The algorithm finds that the category A00- *Cholera *is mapped to the concept *Cholera *and the concept *Cholera *is therefore added to the mapped set. All ancestors to the concept *Cholera *are then also added into the mapped set. The resulting mapped set consists of the concepts shown in black rectangles in Figure 2. (This step is the same as in the multiple chapter division algorithm.)

The additional attributes algorithm uses the mapped set to follow each defining attribute relationship and include all the target concepts in different attribute value set according to which attribute type they are related. The referred concepts shown as blue shaded rectangles in the left of Figure 2 are included in the value set of type *Causative agent*. The referred concepts shown as blue shaded rectangles in the upper right area of Figure 2 are included in the value set of type *Finding site*. The referred concept *Transudate *shown as a blue shaded rectangle in the lower right area of Figure 2 is included in the value set of type *Associated morphology*. Then, in each value set, the concepts that are supertypes of other concepts in the same value set are removed from the value set. In the value set of type *Causative agent*, only the concept *Vibrio cholerae *is left, in the value set of type *Finding site *only the concept *Intestinal structure *remains and the value set of type *Associated morphology *only contains one concept so that value set is unchanged. The category A00- *Cholera *then is assumed to have an attribute of type *Causative agent *with value *Vibrio cholera*, an attribute of type *Finding site *with value *Intestinal structure *and an attribute of type *Associated morphology *with value *Transudate*.

Even if many categories have attributes of a specific attribute type, the usefulness of these attributes can be of limited value if many attributes share the same attribute value. For example, it is of limited use to know that most categories have attributes of the attribute type *Finding site *with the attribute value *Body structure*. We measure the distribution of the attribute values as the proportion of categories that relate attributes of the same attribute type to the same attribute value.

### Fully defined and primitive ancestors

The quality of the multiple chapter division and additional attributes is dependent on how completely modelled the concepts that are mapped from KSH97-P's categories and these concept's ancestors are. (These concepts are the concepts in the mapped sets.) The mapped concepts and their ancestors are therefore extracted and the number of fully defined concepts and primitive concepts are counted. The numbers of outgoing defining relationships from fully defined and primitive concepts are also counted. The proportion of fully defined concepts in SNOMED CT in total are also counted.

Examples of fully defined concepts and primitive concepts are

• The concept *Digestive system finding *is a fully defined concept and is therefore fully defined by its defining relationships' types and targets listed below

○ *Finding site*; *Structure of digestive system*

○ *Is a*; *Finding by site*

• The concept *Accidental poisoning *is a primitive concept and is therefore not fully defined by its defining relationships' types and targets listed below

○ *Is a*; *Poisoning*

• The concept *Cholera *is a fully defined concept and is therefore fully defined by its defining relationships' types and targets listed below

○ *Associated morphology*; *Transudate*

○ *Causative agent*; *Vibrio cholerae*

○ *Finding site*; *Intestinal structure*

○ *Is a*; *Infection due to Vibrio*

○ *Is a*; *Intestinal infectious disease due to Gram-negative bacteria*

### Computational environment

The computational methods described above are performed in a relational database management system (PostgreSQL). SNOMED CT, KSH97-P and the mappings are stored in tables and the computations are executed by SQL queries.

## Results

### Mappings update

#### Release conversion

Six of the category mappings map to inactive concept. All six inactive concepts have historical relationships that relate the inactive concepts to active concepts. Five inactive concepts are each related to one active concept and these active concepts are chosen as replacements for the inactive concepts. One active concept is related to two active concepts and one of these active concepts is chosen as replacement for the inactive concept.

#### Manual comparison of category and chapter mappings

An adequate chapter mapping is a mapping that follows the mapping rules, demands good concordance between the rubric meaning and the concept's meaning in SNOMED CT and a reasonable concordance in SNOMED CT's structure between the chapter mapping and category mappings.

Chapters II, VII and XII, have one mapped concept replaced with one more general concept to cover more of the content in the KSH97-P chapters. In chapter II, the new concept also covers Haemangioma and Lipoma, in chapter VII the new concept also covers vision disorders and in chapter XII the new concept covers diseases and findings in nails and hair.

Chapter XIX has three of the four mappings replaced by one new mapping that more precisely captures the meaning of the chapter categories -- to *accidental *poisoning and traumatic injuries. Chapter III has one mapping added that had not been found earlier through the manual browsing.

The category G72-P *Primary disorder of muscle, unspecified *has its mapping updated to a concept that better grasps the category meaning. The category Q899P *Other specified congenital malformations *has its mapping extended with a disorder concept, because it could be considered a disorder or a morphology anomaly. The category D36- *Benign neoplasm of other and unspecified sites *has its mapping updated from a morphology concept to a disorder concept because of the mapping rule of using disorder or finding concepts prior to morphology concepts. There were two motives for this priority in the mapping rules. Firstly, the disorder and finding concepts match the aim of the classifications KSH97-P and ICD-10 to represent clinical disorders and health problems. Secondly, many of the concepts of interest in SNOMED CT were present both as disorder and morphologic abnormality concepts and instead of choosing both, we decided to give priority to the disorder concept.

#### Statistical chapter mapping

The algorithm for creating statistical chapter mapping created mappings for all chapters.

#### Comparison of manual and statistical chapter mappings

The comparison of manual and statistical chapter mappings gives the following results.

For chapters I, II, V, VI, IX, X, XI, XII, XV and XVII, the manual and the best statistical mappings are mapped to the same concept. For chapters XVIII and XXI, there is no manual mapping. The best statistical mapping for chapter XVIII is *Clinical history and observation findings*, and for chapter XXI is *Procedure*. These concepts are very general and are therefore of no use. These 12 manual chapter mappings are therefore not updated.

For chapters VII, VIII and XIV, the best statistical mappings are mapped to a finding concept. Furthermore, the manual mappings and the second or the third best statistical mappings are mapped to the corresponding disorder concept. After inspection, the manual mappings for chapters VII and VIII are left without changes. The reason is that there are small differences between the numbers of covered categories for the different chapter mappings and the differences consist mainly of non-disease categories. The manual mapping for chapter XIV is changed to the best statistical mapping to better cover its disease categories.

Each of the chapters III, IV, XIII and XIX has manual mappings to more than one concept. For each chapter at least one mapping is equal to a highly ranked statistical mapping and at least one mapping is equal to a poorly ranked statistical mapping. A review shows that categories covered by poorly ranked manual mappings are KSH97-P categories that cover a broad area of relevant diseases and are equivalent to many categories in ICD-10. The manual mappings are therefore considered to be correct and are left without changes.

Chapter XVI has only one manual mapping and that mapping is equal to a poorly ranked statistical mapping. The mapping is to the concept *Perinatal finding*, which covers only one category from the chapter in KSH97-P. The highest ranked statistical mapping is to the concept *Disorder of foetus or newborn*, which covers the other 15 categories in the chapter. A first impression could be that an *Is a *relation from *Disorder of foetus or newborn *to *Perinatal finding *is missing in SNOMED CT. However, the foetus period starts before the perinatal period and *Disorder of foetus or newborn *therefore covers disorders earlier in the pregnancy than *Perinatal finding*. The *Is a*-relationship is therefore not missing, and a manual chapter mapping is added to the concept *Disorder of foetus or newborn*.

### Multiple chapter division

The multiple chapter division is summarised in Table 3, which shows the proportions of categories from each KSH97-P chapter that are divided into new chapters. Some categories are excluded from the multiple chapter division, which can be seen in Table 1. One reason is the lack of mappings from categories and chapters to SNOMED CT, which also can be seen in Table 1. Table 4 shows some examples of how categories are divided in multiple chapters.

**Table 3:** Multiple chapter division summary

Multiple chapter division chapter	Original chapter																				
	
	I	II	III	IV	V	VI	VII	VIII	IX	X	XI	XII	XIII	XIV	XV	XVI	XVII	XVIII	XIX	XXI	All
I	.94									.11	.02	.05	.01	.03	.07	.19				.03	.10

II		1.00										.05			.03		.03				.09

III		.01	.92			.03			.02	.08		.03				.06	.03	.02			.02

IV		.06		.87			.03						.01		.03	.06		.01			.04

V					.91	.06						.02		.02							.05

VI	.04	.04		.03	.19	.86	.08	.05	.10				.01			.06	.13	.02	.02		.07

VII	.01	.03					.97						.01			.06	.03		.03		.04

VIII		.03						.86									.03		.03		.02

IX	.01				.02	.06	.03		.86		.02	.03	.01	.02	.03	.06	.05	.02			.06

X	.02	.06							.04	1.00						.06	.05	.03	.02		.06

XI	.13	.22		.17					.04	.16	.86	.02	.01	.02	.03		.20	.03	.02		.10

XII	.18	.04					.06					.88		.02			.10	.02			.09

XIII	.01	.04				.06		.05		.05	.06	.03	.76	.02	.07		.10		.55		.12

XIV	.04	.25		.10	.02				.04					.83	.10		.13	.09	.02		.10

XV					.02										.90						.03

XVI																1.00	.03				.02

XVII				.03						.03			.01		.03		.90				.04

XVIII																					

XIX	.01				.02				.02		.02	.02	.06		.03	.13		.01	.73		.06

XXI																					

The multiple chapter division is summarised in this table. KSH97-P's original chapters are represented in the columns to the left and the complete KSH97-P in the column furthest to the right. The rows then show the proportion of categories that after the multiple chapter division appear in each chapter. For instance, 11% of categories in chapter X are assigned to chapter I.

As can be seen in Table 1, not all categories are included in the multiple chapter division. One reason is that some categories and chapters are not mapped to SNOMED CT. Another reason is that SNOMED CT's structure does not include all categories in the division.

The multiple chapter division does not intentionally care about the excludes remarks in ICD-10.

**Table 4:** Examples of multiple chapter division

Original chapter number	Category code	Category term	Mapped chapter number
V	F01-	Vascular dementia	V

V	F01-	Vascular dementia	VI

V	F01-	Vascular dementia	IX

V	F01-	Vascular dementia	XIX

IX	I84-	Haemorrhoids	IX

IX	I84-	Haemorrhoids	XI

X	J36-	Peritonsillar abscess	X

X	J36-	Peritonsillar abscess	XI

The first three columns show the KSH97-P's category's chapter number, code and term. The last column shows the category's chapter number according to the multiple chapter division. These categories are only a small selection of examples from the complete multiple chapter division.

### Additional attributes

Table 5 shows the proportion of categories in each KSH97-P chapter and the total that have additional attributes of different attribute types. Table 6 shows the average number of categories that have the same attribute value for each attribute type. The corresponding median values are also calculated in the study and are in most cases close to the average values. Table 7 demonstrates some examples of categories and their additional attributes.

**Table 5:** Attribute types in each chapter

Attribute type	Chapters in KSH97-P																				
	
	I	II	III	IV	V	VI	VII	VIII	IX	X	XI	XII	XIII	XIV	XV	XVI	XVII	XVIII	XIX	XXI	All
After	.04			.03		.03			.02		.02	.02							.03		.01

Associated finding																				.03	.00

Associated morphology	.20	1.00	.15	.13	.02	.26	.61	.52	.44	.74	.45	.73	.57	.55	.17	.38	.93	.11	.81		.47

Associated with			.23	.07			.03			.03		.02						.01	.08		.01

Causative agent	.96			.03	.15	.03		.05		.16	.08	.16	.01	.03	.07	.19	.03		.09	.03	.13

Clinical course	.04				.04	.03	.03	.19	.06	.26	.04		.01	.02			.03	.01	.02		.03

Direct device																				.03	.00

Direct substance																				.03	.00

Due to			.23	.03			.03			.05		.13							.02		.02

Finding context																		.04		.03	.01

Finding informer																		.03			.00

Finding method																	.03	.09		.03	.01

Finding site	.46	.84	.23	.67	.21	.97	1.00	1.00	.94	1.00	.98	.95	.95	.92	.33	.44	.93	.65	.72		.74

Has definitional manifestation	.10		.92	.07	.19	.20		.10	.06	.03	.02	.06	.02	.03	.03	.06	.03	.03			.06

Has focus																				.11	.00

Has intent																				.03	.00

Has interpretation							.25							.03				.10			.02

Interprets					.04	.06	.25	.14				.02	.02	.08			.03	.35		.09	.06

Method																				.40	.01

Occurrence				.03	.02					.03			.04		.07	.63	.90				.06

Pathological process						.03						.02									.00

Procedure context																				.03	.00

Procedure device																				.03	.00

Procedure site																				.06	.00

Procedure site - Indirect																				.03	.00

Subject relationship context																		.04		.06	.01

Temporal context																		.04		.06	.01

The proportion of categories in each chapter and in total that are modelled using each attribute type according to the additional attribute method.

**Table 6:** Different attribute values in each chapter

Attribute type	Chapters in KSH97-P																				
	
	I	II	III	IV	V	VI	VII	VIII	IX	X	XI	XII	XIII	XIV	XV	XVI	XVII	XVIII	XIX	XXI	All
After	1.5			1.0		1.0			1.0		1.0	1.0							1.0		1.3

Associated finding																				1.0	1.0

Associated morphology	1.5	3.8	.7	1.3	.5	1.0	1.4	1.4	1.7	1.8	1.5	1.5	1.7	1.6	1.0	1.5	2.1	1.3	3.7		2.7

Associated with			3.0	1.0			1.0			1.0		1.0						1.0	1.7		1.4

Causative agent	1.1			1.0	1.8	.5		1.0		1.0	1.0	1.0	1.0	2.0	2.0	3.0	1.0		1.0	1.0	1.3

Clinical course	3.0				1.0	1.0	1.0	2.0	3.0	5.0	1.0		1.0	1.0			1.0	1.0	1.0		1.3

Direct device																				1.0	1.0

Direct substance																				1.0	1.0

Due to			1.0	1.0			1.0			.7		1.6							1.0		1.5

Finding context																		4.0		1.0	5.0

Finding informer																		3.0			3.0

Finding method																	1.0	4.0		1.0	3.3

Finding site	1.7	1.3	3.0	1.8	2.0	1.3	1.6	1.9	1.3	1.6	1.4	3.6	1.1	1.7	1.0	.9	1.0	1.4	.9		2.0

Has definitional manifestation	4.0		1.3	1.0	1.8	1.8		1.0	1.5	1.0	1.0	1.3	2.0	2.0	1.0	.3	1.0	.8			2.0

Has focus																				1.3	1.3

Has intent																				1.0	1.0

Has interpretation							4.5							2.0				1.8			4.0

Interprets					.5	.7	3.0	1.5				1.0	1.0	1.0			1.0	.9		1.0	1.2

Method																				2.0	2.0

Occurrence				1.0	1.0					1.0			1.0		1.0	3.3	36.0				9.0

Pathological process						1.0						1.0									2.0

Procedure context																				1.0	1.0

Procedure device																				1.0	1.0

Procedure site																				1.0	1.0

Procedure site - Indirect																				1.0	1.0

Subject relationship context																		4.0		1.0	3.0

Temporal context																		4.0		1.0	3.0

The number of categories, in each chapter and in total, for which each attribute type is used, divided by the number of unique attribute values for each attribute type. This means the table shows the average number of categories that have the same attribute value for each attribute type. The corresponding median values are also calculated in the study and are in most cases close to the average values, but the median values are left out from the paper to shorten the paper.

For example, 66 of the 79 categories in chapter II *Neoplasms *have attributes of type *Finding site *with 49 different attribute values. This means that the corresponding value in the table is 66/49 = 1.3. If, hypothetically, all 79 categories in the chapter have had attributes of type *Finding site *with attribute value *Body structure*, the corresponding value would be 79/1 = 79. In the actual case the attribute information is informative, but in the hypothetical case the attribute information is of limited use.

If few categories have attributes of a specific attribute type in a specific chapter these categories can easily have unique attribute values for these attributes. Therefore it is not useful to compare values in Table 6 for attribute types and chapters where Table 5 shows that only a few categories have attributes.

**Table 7:** Examples of additional attributes

Chapter number	Category code	Category term	Attribute type	Attribute value
VIII	H669P	Otitis media, unspecified	Associated morphology	Inflammation

VIII	H669P	Otitis media, unspecified	Finding site	Middle ear structure

X	J06-P	Acute upper respiratory infections of multiple and unspecified sites	Causative agent	Infectious agent

X	J06-P	Acute upper respiratory infections of multiple and unspecified sites	Clinical course	Sudden onset AND/OR short duration

X	J06-P	Acute upper respiratory infections of multiple and unspecified sites	Finding site	Structure of multiple topographic sites

X	J06-P	Acute upper respiratory infections of multiple and unspecified sites	Finding site	Upper respiratory tract structure

XIII	M259P	Joint disorder	Finding site	Joint structure

The first three columns show a category's chapter number, code and term. The last two columns show the attribute types and attribute values according to SNOMED CT's defining attribute relationships. These categories are only a small selection of examples from the complete additional attributes.

### Fully defined ancestors

Among the concepts that are mapped from KSH97-P's categories and these concepts' ancestors, 1,786 (63%) concepts are fully defined and 1,061 (37%) are primitive. There are a total of 10,010 outgoing defining relationships from the concepts and their ancestors. From all concepts in SNOMED CT, 13% are fully defined.

## Discussion

### Multiple chapter division

#### Chapter priorities

Most categories are included in their own original chapter, which reflects the structure of KSH97-P and SNOMED CT and the intention of the mappings. The lack of inclusions in chapters XVIII and XXI are explained by the lack of chapter mappings for these chapters.

The largest inclusion of categories in a chapter other than the original is the inclusion of 55% of the categories of chapter XIX *Injury, poisoning and certain other consequences of external causes *in chapter XIII *Diseases of the musculoskeletal system and connective tissue*. As seen in Table 2, chapter XIX has priority over chapter XIII according to the excludes remarks in ICD-10, which implies that those categories from chapter XIX would also probably be fitting for chapter XIII. In other words, external causes often injure the musculoskeletal system and connective tissues, which seems reasonable.

Chapter II *Neoplasms *has many inclusions of its categories in chapter XIV *Diseases of the genitourinary system *and chapter XI *Diseases of the digestive system *and smaller inclusions in eight other chapters (see Table 3). In addition, according to the excludes remarks, chapter II in ICD-10 results in having priority over all chapters where it has inclusions, except for chapter IV (see Table 2). Thus neoplasms can be ordered both according to the fact they are neoplasms and according to the affected body site. Most other inclusions of categories in chapters other than the original can be explained analogously by chapter priorities.

The biggest exceptions where inclusions of categories in other chapters cannot be explained by the excludes remarks' priorities are

• 19% of the categories in chapter V *Mental and behavioural disorders *are included in chapter VI *Diseases of the nervous system*

• 19% of the categories in chapter XVI *Certain conditions originating in the perinatal period *are included in chapter I *Certain infectious and parasitic diseases*

• 16% of the categories in chapter X *Diseases of the respiratory system *are included in chapter XI *Diseases of the digestive system*

• 10% of the categories in chapter IX *Diseases of the circulatory system *are included in chapter VI *Diseases of the nervous system*.

Further analysis of these exceptions shows these categories fit into the other chapters although there are no excludes remarks' priorities that can explain the inclusions. In addition, Table 3 shows that many chapters have small fractions of their categories included in other chapters. Evidently, chapter division is a complex task that cannot be accomplished through a set of simple excludes remarks.

#### Omitted categories

As Table 1 reveals, not all categories are included in the multiple chapter division. One reason is that some categories and chapters are not mapped to SNOMED CT's concepts. If all categories and chapters had been possible to map, more categories would probably have been included in the multiple chapter division. Another reason is that SNOMED CT's structure does not include all categories in the division. However, the exclusion of categories in the multiple chapter division because of SNOMED CT's structure does not necessarily mean that its structure is incomplete. The reason is that some categories do not fit into any chapter. Due to pragmatism, these categories have been included in the chapters because they were needed in KSH97-P and all categories must be included in a chapter. One example is the KSH97-P category D86- *Sarcoidosis *in KSH97-P chapter III *Diseases of the blood and blood-forming organs and certain disorders involving the immune mechanism*, which according to SNOMED CT's structure is a *Multisystem disorder*.

#### Clinical meaning

The multiple chapter division shows that the chapter division in KSH97-P, which only allows one category to be included in one chapter, hides information about the categories in KSH97-P. It also shows that it is possible to extend the chapter division to a multiple chapter division using mappings to SNOMED CT and SNOMED CT's poly-hierarchical *Is a *relations. Because of the similarities in structure between KSH97-P and ICD-10, it is likely that the same condition applies to ICD-10 and in other similarly structured medical terminology systems.

This hidden information about categories in KSH97-P explored by our mapping consists of multiple consistent views useful to clinicians for a range of purposes. It is an illustrative example of the advantages of a poly-hierarchy in a context impossible to represent within a mono-hierarchy [6]. Clinically relevant consistent views can be used for navigation to support classification, possibly improving coding validity and reliability. Moreover, such views can be a base for multipurpose data aggregation, an area where the structure of ICD-10 has shown limitations due to limitations in its chapter structure [36]. A small selection of examples from the complete multiple chapter division is shown in Table 4. For example, F01- *Vascular dementia *is mapped to four original chapters. An example of applying the multiple chapter division method on 2.5 million primary health care encounters is given by Vikström et al. [37]. Finally, such multiple chapter division can be used to support navigation in clinical information retrieval systems and decision support systems.

### Additional attributes

#### Attribute types

As can be seen in Table 5, the attribute type *Finding site *is used for 74% of the categories in KSH97-P and *Associated morphology *for 47% of the categories in KSH97-P. These attributes are therefore suitable to use for general analysis of KSH97-P and also for adding multiple hierarchies based on *Finding site *and *Associated morphology *to KSH97-P. Further analysis shows that most categories that can occur only during a specific period of life have the attribute type *Occurrence *associated with them and categories that can occur during any period of life have not. Despite *Occurrence *being used for only 6% of all categories in KSH97-P (as seen in Table 5), it is therefore still useful for general analysis of KSH97-P.

Table 5 also shows that--except for *Finding site *and *Associated morphology*--there are no attribute types frequently used for all categories in KSH97-P. However, other attribute types are useful for analysis of specific chapters in KSH97-P. For example, *Causative agent *is used for 96% of the categories in chapter I and *Has definitional manifestation *is used for 92% of the categories in chapter III.

In comparison with the earlier work presented in Petersson et al. [25] and Nilsson et al. [34], the attribute location in the earlier work is similar to the attribute type *Finding site *in this study. The attributes origin and type in the earlier work are not found in this study and the reason is the very general meaning of these two attributes.

#### Attribute values

According to Table 6, most attribute types only relate one or a few categories to each attribute value. For example, the commonly used attribute types *Finding site *and *Associated morphology *relate 2.0 and 2.7 categories to each attribute value respectively. In chapter I, *Causative agent *relates 1.1 categories to each attribute value, and in chapter III, *Has definitional manifestation *relates 1.3 categories to each attribute value. Thus, attribute values are specific to one or a few categories and not general for many categories. For *Occurrence*, many categories relate to the same attribute value, which is due to the fact many categories represent events occurring during the same period of life and not due to the use of too general attribute values.

If few categories have attributes of a specific attribute type in a specific chapter these categories can easily have unique attribute values for these attributes. Therefore it is not useful to compare values in Table 6 for attribute types and chapters where Table 5 shows that only a few categories have attributes.

#### Attributes in different chapters

When combined, the information from Table 5 and Table 6 would reflect the chapter structure of ICD-10, indicative of the quality of the semantic enrichment of KSH97-P. In essence, three patterns, described below, of well-represented relationships appear, i.e. attribute types used for a large proportion (above an arbitrarily chosen threshold of 90%) of categories in a chapter (Table 6) and attribute values that to a high degree are exclusive in a chapter (Table 6). These patterns reflect the multidimensional structure of ICD-10.

First, chapter I *Certain infectious and parasitic diseases *is appropriately described through the attribute type *Causative agent*. Second, the attribute type *Associated morphology *signifies chapter II *Neoplasms *and chapter XVII *Congenital malformations, deformations and chromosomal abnormalities*. Both of them are also reasonably well-described (although less than 90%) through the *Finding site *attribute type. Third, all organ system chapters except for chapter III *Diseases of the blood and blood-forming organs and certain disorders involving the immune mechanism*, chapter IV *Endocrine, nutritional and metabolic diseases*, and chapter V *Mental and behavioural disorders *include *Finding site *attributes.

Chapter XIX *Symptoms, signs and abnormal clinical and laboratory findings, not elsewhere classified *could have been expected to belong to the second pattern, but fell short of our admittedly arbitrary 90% threshold. Ideally, as a supplement to the disease-oriented chapters, these categories would also be well-described with respect to finding site, but they are not.

Regarding *Finding site*, two organ system chapters stand out as particularly ill-defined: chapter III and chapter V with 23% and 21% of categories respectively. The chapter III categories, whose pragmatic classification is recognized by WHO on page 13 in [7], mainly consist of deficiencies and anaemias. These are characterised through *Has definitional manifestation *(92% of categories) whereas chapter V does not have a signifying attribute type. In chapter IV, *Finding site *is still the most frequently used attribute type (67%), while chapter V does not have a signifying attribute type. This indicates that something is missing in the resulting additional attribute model, but a more thorough analysis of the underlying mechanisms is beyond the scope of this study.

We also lack proper attribute additions for chapters XV *Pregnancy, childbirth and the puerperium*, and XVI *Certain conditions originating in the perinatal period*. Since *Occurrence *refers to a specific period of life during which a condition first presents [30], it would be a suitable attribute type here. On the other hand, 36 out of 40 congenital conditions (chapter XVII) refer to the same occurrence attribute. The occurrence attribute does occur in 63% of the categories in chapter XVI though.

Due to the diversity of chapter XVIII *Symptoms, signs and abnormal clinical and laboratory findings, not elsewhere classified *[38], it is not surprising that its categories are inconsistently described throughout the mapping. Exploring the soundness of these attributes is also beyond the scope of this study. In addition, this chapter and chapter XXI *Factors influencing health status and contact with health services *were excluded from chapter mapping. Based on the lack of overall pattern in added attributes, the initial remark regarding the difficulty of chapter mapping--i.e. chapter rubrics referring to heterogeneous collections rather than clinical concepts--proved reasonable.

Two things concerning this analysis must be pointed out. First, a combination of an attribute type and an attribute value does not necessarily have to be unique; rather, a set of such relationships would define a category. For example, both categories A00- *Cholera *and A03- *Shigellosis *have a *Finding site *of *Intestinal structure*. However they are uniquely defined, because A00- *Cholera *has a *Causative agent *of *Vibrio cholera *and A03- *Shigellosis *has a *Causative agent *of *Shigella*. Second, the uniqueness numbers given in Table 6 are mean values and in addition median values are studied without providing further information. Variations in distribution among chapters might be a source of error and it would be interesting to analyse this in the future. However, the general picture lines up fairly well with the following chapter pattern, which to some extent is evident in the chapter rubrics:

• the essential chapter intention

• phenomena closely related to the (essential) chapter intention

• other phenomena, less closely related to the (essential) chapter intention

#### Clinical meaning

Additional attributes can be discussed from several perspectives. From a clinical point of view, attributes concerning *Finding site*, *Associated morphology *and *Causative agent *can be considered useful complements to the traditional classifications. In future systems for coding of patient data and data aggregation, these attributes can be highly valuable. The attribute type *Finding site *includes a number of clinically relevant attribute values such as *Upper respiratory tract structure*, *Head and neck structure *and *Joint structure *which can be considered of high interest in health planning and epidemiology. These kinds of data aggregations were previously not easily available.

Furthermore, the attribute type *Associated morphology *includes a long list of clinically relevant attribute values for data aggregation. For instance, *Traumatic abnormality *and *Inflammation *can improve data aggregation of the clinical content in electronic health records, and both attribute values should be of interest for health planning as well as quality improvement. Further, the attribute type *Causative agent *includes attribute values such as *Infectious agent *(in general), *Bacteria*, and *Tobacco *that are common and important causes to health problems that given our mapping can be analysed in a broad and more reliable way than before. Several other attribute types as seen in Table 6 and their underlying lists of specific attributes give a broad system of clinical relevant attributes that needs to be further explored and evaluated in future research. An example of applying the additional attributes method on 2.5 million primary health care encounters is given by Vikström et al. [37].

### Fully defined ancestors

The fact that 37% of the used SNOMED CT concepts are primitive has most likely decreased the number of additional attributes and maybe also decreased the number of multiple chapters, but it is difficult to estimate how much. It does not mean that 37% of the attributes and chapters are missing because even primitive concepts have defining relationships. The effects are also dependant on where the primitive concepts are placed in SNOMED CT's structure. The only thing the numbers reveal is at least 1,061 defining relationships should be added to the existing 10,010 defining relationships used to make the concepts fully defined. This is because all primitive concepts lack at least one defining relationship to be fully defined and the number of used primitive concepts is 1,061.

It is beneficial that 63% of the used concepts are fully defined when the fraction of fully defined concepts of the entire SNOMED CT is 13%. However, the used concepts are central for Swedish primary health care and probably in primary health care in many other countries as well. It would be interesting to explore the possibilities to increase the number of fully defined concepts in this subset.

### Enriching classifications

According to the meta classification by Rossi Mori et al. [1], KSH97-P is a first generation system, but after the additions of the multiple chapter division and additional attributes, it is possible to compare KSH97-P with a second generation system, at least with respect to structure. The categories in KSH97-P constitute a list of categories, and the additional attributes and their structure in SNOMED CT can be seen as a cross-thesaurus. The relationships between KSH97-P categories and their attributes correspond to a knowledge base of dissections. SNOMED CT's concept model together with information about how the mappings are done can be seen as a categorical structure.

One major improvement of a second generation system in comparison with a first generation system is, according to Rossi Mori et al., the possibility of automatically rearranging categories to satisfy different purposes [1]. This study shows that KSH97-P's categories can be rearranged using the multiple chapter division and prioritisation to include concepts in other chapters than they normally are. It is also highly possible to rearrange the categories using the additional attributes according to *Finding site *and to a lesser extent according to *Associated morphology*. Even more reorganisations would probably have been possible if a higher proportion of the used concepts in SNOMED CT were fully defined.

Cimino [2] advocates similar characteristics as Rossi Mori et al. [1]. Four desired characteristics according to Cimino are poly-hierarchy, formal definitions, multiple granularity and multiple consistent views [2]. SNOMED CT fulfills these criteria, but none of them is fulfilled by KSH97-P. KSH97-P's "hierarchy" of chapters and categories does not fulfil what Cimino [2] terms multiple granularity; the reason is that only categories are meant to be used for coding. In ICD-10, on the other hand, the possibility of coding on three, four or in some parts five-character level would qualify as multiple granularity. However, this study shows that it is possible to use SNOMED CT to enrich KSH97-P in such a way that it fulfills Cimino's characteristics of poly-hierarchy, formal definitions, multiple granularity and multiple consistent views.

The formal definitions of SNOMED CT concepts make it possible to automatically perform the rearrangements of the categories in this study. The mappings from KSH97-P categories to SNOMED CT concepts, together with SNOMED CT concepts' formal definitions may also, to some degree, be seen as formal definitions of KSH97-P categories. SNOMED CT poly-hierarchy is used for constructing the multiple chapter division. The multiple chapter division also turns KSH97-P into a poly-hierarchical terminology system. SNOMED CT multiple granularity makes it possible to map both KSH97-P's chapters and categories to SNOMED CT concepts, and it also makes new multi-granular aggregations of KSH97-P's categories possible. SNOMED CT's multiple consistent views are used for generating both the multiple chapter division and the additional attributes. The possibility of showing KSH97-P categories according to SNOMED CT structure together with the multiple chapter division or different attributes in the additional attributes makes it possible to show KSH97-P in multiple consistent views.

A result of this study is that it is possible to keep the mono-hierarchical medical terminology system KSH97-P as it is and use it as an aggregation terminology, but at the same time enrich it with a more complete model of health problems. This can be an integrated way for medical terminology systems with different purposes and characteristics to co-exist, which Straub et al. speak in favour of [6]. However, this study also shows that since mono-hierarchical medical terminology systems give a limited view of the real world, a better solution in the long run is to use terminology systems with richer structures, but with integrated aggregation structures for statistical calculations.

### Rearranging KSH97-P categories

The methods presented in this study can be used to group KSH97-P categories together in groups according to different criteria than KSH97-P's and ICD-10's inherited design considerations' states. In other words, KSH97-P categories can be seen in multiple consistent views.

For example, according to the design considerations, KSH97-P's original chapter I *Certain infectious and parasitic diseases *only contains certain, and not all, infectious and parasitic diseases. However, using the multiple chapter division it is possible to include 17 categories that are not included in the original chapter in the group. Examples of these categories are J22-P *Acute lower respiratory infection*, L01- *Impetigo *and O86-P *Puerperal infections*. The method also proposes to exclude 5 categories from the original chapter in the group. Examples of these categories are A09-P *Diarrhoea and gastroenteritis of presumed infectious origin *and B94-P *Sequelae of other specified infectious and parasitic diseases*.

The resulting grouping of infectious and parasitic diseases categories is also an example of a possibility to change the chapters' priorities, given by the chapters' excludes remarks, in KSH97-P's mono-hierarchical design. The highest prioritised chapters include all relevant categories for that chapter, but lower prioritised chapters have only the relevant categories not included in a higher prioritised chapter. Collecting all categories related to infectious and parasitic diseases therefore gives the chapter the highest priority.

In the same way KSH97-P's original chapter XI *Diseases of the digestive system *only contains some and not all digestive system diseases. However, using the multiple attributes and including all categories that have an attribute type of *Finding site *with an attribute value of any subtype of *Structure of digestive system *it is possible to include 65 categories that are not included in the original chapter in the group. Examples of these categories are A00- *Cholera*, C16- *Malignant neoplasm of stomach *and R13- *Dysphagia*. The method also proposes to exclude 7 categories from the original chapter in the group. Examples of these categories are K42- *Umbilical hernia *and K65- *Peritonitis*.

It is also possible to use the methods described above to make groups according to other criteria than emulating an original chapter in KSH97-P. For example, suitable categories to include in a group related to the upper respiratory tract can be found by collecting all categories that have an attribute type of *Finding site *with an attribute value of any subtype of *Upper respiratory tract *or the concept itself. 26 categories fulfil this criterion and examples are C32- *Malignant neoplasm of larynx*, J32- *Chronic sinusitis *and J342 *Deviated nasal septum*. A similar method can be used to find categories related to the lower respiratory tract and 16 categories fulfil this criterion. Examples are C34-P *Malignant neoplasm of trachea, bronchus and lung*, J18-P *Pneumonia *and J42-P *Chronic bronchitis*. This can be seen as introducing multiple granularities into KSH97-P.

### Mapping versus modelling

One way of looking at the difference between second and third generation terminologies is the discrepancy between mapping and modelling, that is, a matter of descriptions and definitions. Information reuse, which is the underlying topic of this paper, is always restrained by the limitations of the organising principles behind the vocabulary in which it was originally stored. Semantically interoperable information systems, e.g. clinical decision support tools connected to electronic health records, require a higher degree of the above: modelling in the sense of definitions, but a reasonable assumption is that a great deal of the vocabulary underlying all currently available information will never be modelled in this fashion.

Instead of using mapping methods in this study it would have been possible to extend KSH97-P with multiple chapters and additional attributes using entirely manual modelling as in the ICD-10 projects mentioned in the Background. It is possible the results may have been of higher quality than that of the mapped ones, but the modelling would have required more resources to perform than the mapping. The difference in used resources would have been even higher if larger medical terminology systems, like ICD-10, were extended with multiple chapters and additional attributes. Furthermore, as opposed to providing describing attributes, the definition of ICD categories seems to be troubled with difficulties regarding pragmatic, implicit and even unclear semantics. Depending on the purpose and the intended use and reuse of coded information, we believe enrichment through mapping might be an alternative to reconstruction when it comes to meeting requirements on new versions of classifications such as ICD and DSM.

Besides relying on descriptions rather than definitions, mapping imposes an extra layer of uncertainty and source of error. In this study, findings such that some categories did not make their way to their original chapters and that not all categories received additional attributes as expected could be explained by

• KSH97-P (source) issues such as counter-intuitive and overly pragmatic chapter inclusion

• Mapping problems (which are bound to occur when diverse contexts meet)

• SNOMED CT (target) problems such as incomplete or even faulty relationships

### Statistical chapter mapping

The algorithm for the creation of statistical chapter mapping was designed during this study. The premise for the algorithm is to prefer chapter mappings to concepts where the concept's descendants are mapping targets of many categories in the same chapter but few categories from other chapters. However, due to the design of KSH97-P, represented by the inherited excludes remarks from ICD-10, some exceptions from this principle must be allowed.

During the algorithm design, different alternatives were considered for the formula that transforms the variables *categories current chapter *(*c*) and *categories other chapters *(*o*) to a ranking score in line with the design assumptions. The initial considerations result in two main alternatives. The alternatives are the unweighted formula  and the weighted formula .

The unweighted formula gives by definition the score 1, the maximum, for all concepts where *o *= 0 and a score < 1 for all concepts where *o *> 0. This characteristic interferes with the design assumption that some categories from other chapters must be tolerated. A test also shows that the formula gives a maximum score to many categories on lower levels in the hierarchy that subsume only one of a few category mapping targets and lower scores to categories on higher levels in the hierarchy that subsume many category mapping targets from a chapter. These characteristics make the unweighted formula unsuitable for the statistical chapter mapping algorithm and the weighted formula is therefore selected.

### Limitations

The results in this study are dependent on the quality of the mappings from KSH97-P to SNOMED CT and the quality of the modelling of the relationships in SNOMED CT. However, actions have been taken to use mappings of good quality. The baseline category mapping and initial chapter mapping are constructed using quality-assurance methods described by Vikström et al. [35]. The study described in this paper also starts with two mapping validation methods where a manual comparison of category and chapter mappings and a comparison of manual and statistical chapter mappings are performed.

A limitation in the manual comparison of category and chapter mappings is the focus on minimising the number of categories that will not belong to any chapter in the multiple chapter division. Other incorrectly mapped categories could therefore have been disregarded.

The methods used during comparison of manual and statistical chapter mappings for finding potential incorrect mappings have one component trying to minimise the number of categories included in other chapters than the original KSH97-P chapter. Thus there is a risk the updates during this comparison remove correct additional chapters in the multiple chapter division. However, the method is only used for finding potentially incorrect mappings and all potentially incorrect mappings are manually inspected before any changes are made to the mappings. The risk of removing correct additional chapters in the multiple chapter division is therefore low. Another limitation is that the statistical chapter mapping's scoring formula was designed during this study and are not well established in this research area. However, the scores for a possible mapping are only used for comparisons with scores for other chapter mappings and the results of the comparisons are only used as a base for the manual inspection.

### Future work

Besides continuing this study with a more detailed look at the semantics on category level, an interesting extension is to analyse the complete ICD-10 and not only a primary health care version. The reason why ICD-10 is not analysed in this study is the lack of mappings from ICD-10 to SNOMED CT, but this may change in the future. The result of a study analysing ICD-10 using these methods could be useful input to the work of revising ICD-10 into ICD-11. For example, the study may provide suggestions of possibly new structures in ICD-11. It would also be interesting to analyse other medical terminology systems using the same methods as in this study.

The mapping provided by the National Health Service in the United Kingdom is not suitable for this kind of study, because it is only a mapping from SNOMED CT to ICD-10 [39]. The focus of that mapping is thereby to correctly map as many SNOMED CT concepts, independent of the concepts' granularity, as possible to ICD-10 categories instead of mapping each ICD-10 category to one or a few SNOMED CT concepts with the same granularity.

## Conclusions

It is possible to use mappings from KSH97-P to SNOMED CT and SNOMED CT's structure to enrich KSH97-P's mono-hierarchical structure. The enrichment makes it possible to group KSH97-P categories in different ways than KSH97-P's original chapter division. These groupings give new and more flexible possibilities to, for example, aggregate diagnostic data for epidemiologic purposes and search for categories to use during coding of the diagnoses in electronic health records.

A new and poly-hierarchical chapter division of KSH97-P categories can be created using the category and chapter mappings and SNOMED CT's generic structure. ICD-10 and KSH97-P have mono-hierarchical chapter divisions and implicit design considerations about in which chapter to include a specific category. The poly-hierarchical chapter division makes these implicit design considerations explicit.

KSH97-P's categories can be extended with attributes using the category mappings and SNOMED CT's defining attribute relationships. The most frequent attribute types in the entire KSH97-P are *Finding site *and *Associated morphology*. However, in single chapters, other attribute types are also frequent. The attribute values are modelled with a high level of detail.

## Glossary

additional attribute: An attribute added to a KSH97-P category using mappings to SNOMED CT and SNOMED CT's structure.

ancestor: Supertype to a concept in a hierarchy.

category: A class in a terminology system such as ICD-10 and (in this paper in most cases) KSH97-P.

category mapping: A mapping from a KSH97-P category to one or more SNOMED CT concepts.

chapter: The most coarsely granulated grouping of categories in terminology systems such as ICD-10 and (in this paper in most cases) KSH97-P.

chapter mapping: A mapping from a KSH97-P chapter to one or more SNOMED CT concepts.

concept: A class in SNOMED CT.

defining attribute relationship: A relationship between a concept and an attribute concept in SNOMED CT. The relationship is always a defining relationship.

defining relationship: A relationship that is used to formally define a SNOMED CT concept.

descendant: Subtype to a concept in a hierarchy.

description: A textual term that describes a SNOMED CT concept and information about the term.

excludes remark: A remark to an ICD-10 chapter informing that the categories in the remark could have been included in the chapter, but are instead included in another chapter(s) due to design considerations.

fully defined concept: A SNOMED CT concept that is formally fully defined by its defining relationships.

generic relationship: A relationship that relates a subtype concept to a supertype concept. In SNOMED CT, the relationship is always a defining relationship. The relationship's type is *Is a*.

historical relationship: Relates an inactive concept to an active concept in SNOMED CT.

multiple chapter division: A division of KSH97-P categories where each category can be included in more than one chapter. The division is created using mappings from KSH97-P to SNOMED CT and SNOMED CT's structure.

navigational concept: A concept in SNOMED CT only intended to simplify navigation in the hierarchies.

original chapter: The chapter a category belongs to according to KSH97-P's original chapter division.

primitive concept: A SNOMED CT concept that is formally partly defined by its defining relationships.

relationship: Relates a concept to another concept in SNOMED CT.

rubric: A textual term that describes a category or chapter.

## Competing interests

MN is a member of IHTSDO's Technical Committee and Sweden's Lead Technical Contact towards IHTSDO. HA is a member of IHTSDO's Implementation and Innovation Committee.

## Authors' contributions

MN participated in the design of the study, designed, implemented and ran the algorithms for the analysis, participated in the analysis and drafted the manuscript. AV participated in the design of the study, created the mappings, participated in the analysis and wrote parts of the manuscript. GHN, HÅ and HÖ participated in the design of the study, helped with the analysis and wrote parts of the manuscript. All authors read and approved the final manuscript.

## Supplementary Material

Additional file 1 **Category mapping**. This file contains the final mapping from KSH97-Pâ€™s categories to SNOMED CTâ€™s concepts. The first column contains the categoriesâ€™ codes and the second column the mapped conceptsâ€™ concept IDs. The first row contains column headings. The file is a text file with the text encoding Unicode (UTF-8). The columns are separated with tab characters and the rows are terminated with carriage return and line feed characters. Click here for file

Additional file 1 **Chapter mapping**. This file contains the final mapping from KSH97-Pâ€™s chapters to SNOMED CTâ€™s concepts. The first column contains the chaptersâ€™ codes and the second column the mapped conceptsâ€™ concept IDs. The first row contains column headings. The file is a text file with the text encoding Unicode (UTF-8). The columns are separated with tab characters and the rows are terminated with carriage return and line feed characters. Click here for file
